# Uneven Terrain Walking with Linear and Angular Momentum Allocation

**DOI:** 10.3390/s23042027

**Published:** 2023-02-10

**Authors:** Zhicheng He, Songhao Piao, Xiaokun Leng, Yucong Wu

**Affiliations:** 1 School of Computer Science and Technology, Harbin Institute of Technology, Harbin 150001, China; 2 Shenzhen Key Laboratory of Biomimetic Robotics and Intelligent Systems, Department of Mechanical and Energy Engineering, Southern University of Science and Techology, Shenzhen 518055, China

**Keywords:** centroidal momentum, momentum allocation, motion control, uneven terrain walking

## Abstract

Uneven terrain walking is hard to achieve for most child-size humanoid robots, as they are unable to accurately detect ground conditions. In order to reduce the demand for ground detection accuracy, a walking control framework based on centroidal momentum allocation is studied in this paper, enabling a child-size humanoid robot to walk on uneven terrain without using ground flatness information. The control framework consists of three controllers: momentum decreasing controller, posture controller, admittance controller. First, the momentum decreasing controller is used to quickly stabilize the robot after disturbance. Then, the posture controller restores the robot posture to adapt to the unknown terrain. Finally, the admittance controller aims to decrease contact impact and adapt the robot to the terrain. Note that the robot uses a mems-based inertial measurement unit (IMU) and joint position encoders to calculate centroidal momentum and use force-sensitive resistors (FSR) on the robot foot to perform admittance control. None of these is a high-cost component. Experiments are conducted to test the proposed framework, including standing posture balancing, structured non-flat ground walking, and soft uneven terrain walking, with a speed of 2.8 s per step, showing the effectiveness of the momentum allocation method.

## 1. Introduction

A biped robot has a high degree of freedom and is expected to achieve stable motion in complex environments. Thus, biped robot motion control is one of the research hotspots in the field of robots. To enable stable walking of a humanoid robot, various zero moment point (ZMP) controllers are designed to track the motion trajectory and resist external force disturbance when the robot is walking [1,2,3,4]. With these ZMP controllers, various humanoid walking gaits are realized with different prototypes [5].

When ZMP controllers are to change the contact force between the robot and the environment, some other controllers focus on changing the contact position of the robot to further improve the anti-interference ability. The capture point control uses the instantaneous capture point to calculate the landing position [6,7] of the foot and take a new step to restore the body to a stable state when the body loses balance. Some research also formulates a nonlinear optimization problem to solve the landing position in real time [8]. Based on contact force control and contact position control, stable and robust humanoid walking is achieved.

However, for child-size biped robots, because of the inability of their joints to perform precise force control, it is difficult for them to precisely control the position of ZMP in the supporting polygon. Moreover, when the ZMP is out of the support polygon, for example when the robot is disturbed by a large external force, these ZMP controllers have little effect on the balance control process. As for contact-position-related methods, the robot needs to quickly adjust the landing position during the stepping process, so the joints needs to have a fast response ability to action commands, which may exceed the performance of most child-size biped robot joints.

Apart from contact force control and contact point control, the balance control of humans and other bipeds also involves the coordinated movement of the upper body. Researchers have also explored balance control methods based on the upper body [9] and centroidal momentum [10,11]. We believe that, for child-size biped robots, because of the limited dynamic response speed of their joints and the inability of their joints to perform precise force control, it is difficult to improve the motion control effect by adjusting the foot contact force or adjusting the foot landing position. However, to adjust the linear and angular centroidal momentum, only the position and velocity trajectory of the robot’s center of mass (CoM) need to be adjusted. So a momentum distribution strategy is more likely to achieve a better anti-disturbance control effect.

He et al. proposed a method to compensate static walking trajectory with angular momentum offline to improve the dynamic characteristics of the whole body trajectory [12]. In this paper, We focus on real-time balancing control and develop a momentum allocation framework. In order to realize momentum allocation, an allocation principle needs to be established. In this work, the allocation principle is to reduce the linear momentum and angular momentum step-by-step in the double-support balancing stage of walking. When the linear momentum is large, the rate of change of angular momentum is allocated to compensate for the linear momentum. Similarly, when the angular momentum is large, the rate of change of linear momentum is used to compensate for the angular momentum. Through continuous compensation control and the effect of the posture controller and the admittance controller, both linear and angular momentum will be gradually reduced to realize balance control.

Briefly, the main contributions of this paper are as follows:

• Providing a momentum allocation method considering the change of centroidal angular and linear momentum during robot movement to increase the stability of robot locomotion.
• Demonstrating the effectiveness of angular momentum compensation and linear momentum compensation.
• Realizing uneven terrain walking in a child-size humanoid Roban.

The rest of this paper describes our approach based on the following sections. In Section 2, we introduce the relationship between Centroidal Moment Pivot (CMP), ZMP, and angular momentum rate. Based on that, we describe the proposed momentum allocation method. Section 3 details the framework with three controllers that we use to realize uneven terrain walking. We show our simulation and experimental results in Section 4 and conclude the paper and describes our future work in Section 5.

## 2. Ground Reference Point and Robot Motion Analysis

ZMP is a widely used ground reference point for biped planning and control. However, when considering the dynamics of multi-rigid bodies, it cannot accurately calculate the trajectory of the robot’s center of mass (CoM) [13,14]. As we know, ZMP indicates the sum of the change of angular momentum and the change of linear momentum of the system [15,16,17], and the change of angular momentum will affect the change of linear momentum. However, the CMP reference point will not be influenced by the whole body angular momentum change on the robot motion, thus a beter ground reference point for planning and control. This section will introduce the difference between ZMP and CMP indicators [18,19] and describe the equation of motion of the centroidal momentum and its rate of change. The notations used in this article are listed in Table 1.

### 2.1. Difference between ZMP and CMP

Popovic et al. discussed the difference between ZMP and CMP [20] and the importance of CMP. For the convenience of readers, the relationship between them is briefly explained. ZMP as a function of the CoM position, net CoM force ($F=M\ddot{x}$), and net moment of the CoM can be expressed as follows in the *x*-direction.

(1) $$x_{ZMP}=x_{CoM}-\frac{F_{Gx}}{F_{Gz}}z_{CoM}-\frac{\tau_{y}\left({\vec{r}}_{CoM}\right)}{F_{Gz}}$$

where $\tau_{y}\left({\vec{r}}_{CoM}\right)$ is the total moment acting on the CoM, and $F_{G}$ is the ground reaction force. In a normal linear inverted pendulum model (LIPM), the height of the center of mass $z_{CoM}$ does not change, and the change of angular momentum of the robot is neglected. Therefore, Equation (1) can be written below:

(2) $$x_{ZMP}=x_{CoM}-\frac{F_{Gx}}{F_{Gz}}z_{CoM}=x_{CoM}-\frac{z_{CoM}}{g}{\ddot{x}}_{CoM}$$

**Figure 1:**
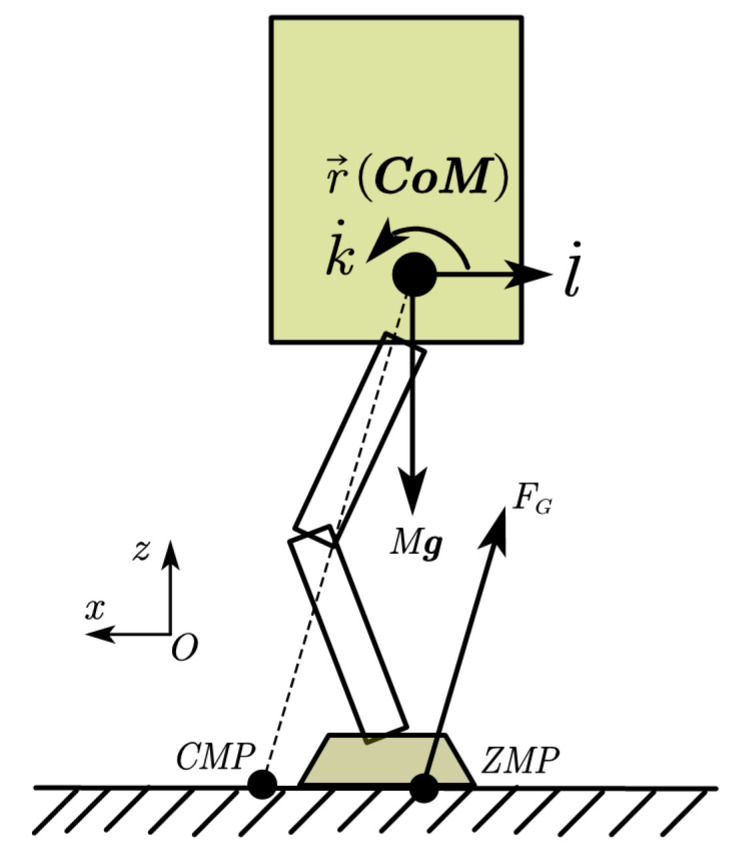
External forces analysis. In the xOz plane, the robot is subjected to gravity force, ground reaction force, and the inertial force of its own acceleration.

The CMP ground reference point was introduced in [21] to discover the relationship between angular centroidal momentum and various postural balance strategies. The CMP is defined as the point where a line intersects with the external contact surface and is parallel to the ground reaction force, passing through the CoM, as shown in Figure 1. It can be expressed as (3).

(3) $$x_{CMP}=x_{CoM}-\frac{F_{Gx}}{F_{Gz}}z_{CoM}$$

The relationship between CoM and ZMP is determined by Equation (2), ignoring the angular momentum of the robot. By taking into account the angular momentum, the relationship between CoM and CMP is determined by Equation (3). Combining Equations (1) and (3), the CMP can be calculated from the ZMP location, the vertical ground reaction force, and the moment of the CoM.

(4) $$x_{CMP}=x_{ZMP}+\frac{\tau_{y}\left({\vec{r}}_{CoM}\right)}{F_{Gz}}$$

### 2.2. Centroidal Momentum Rate of Change

Orin et al. proposed a centroid dynamics model [22]. Using the centroidal momentum matrix(CMM), the centroidal momentum can be easily computed from Equation (5), while the 6×1 centroidal momentum vector $h_{G}={\left[k^{T}\;\;l^{T}\right]}^{T}$ consists of the angular and linear centroidal momenta of the robot, the 6 × n matrix $A_{G}$ is the CMM, and n × 1 $\dot{q}$ is the generalized velocity vector.

(5) $$h_{G}=A_{G}\left(q\right)\dot{q}$$

According to Newton’s laws of motion, the rate of change of angular and linear momentum at the CoM, $\dot{k}$ and $\dot{l}$, is equivalent to the resultant effect of all external forces [23,24]. Figure 1 shows the external forces that act on the standing robot: the ground reaction force $F_{G}$ and the weight Mg of the robot.

Consider the case where only continuous contact wrench acts on the robot. Express the relation between the momentum rate of change and external forces in the sagittal plane (xOz plane) in mathematical equations as follows.

(6) $$\begin{array}{cc} \dot{k} & =\left(x_{CoP}-r_{CoM}\right)\times F_{G}=\tau_{y}\left({\vec{r}}_{CoM}\right)\\ \dot{l} & =Mg+F_{G}=F_{Gx} \end{array}$$

Note that the linear momentum rate of change is determined by the ground reaction force $F_{G}$ only, but the angular momentum rate of change is determined by both the $F_{G}$ and center of pressure (CoP) location.

By inserting Equation (6) into Equation (1), we obtain the relation of the linear momentum rate of change, the angular momentum rate of change, and ZMP, which will be used for momentum allocation control.

(7) $$x_{CoM}-x_{ZMP}=\frac{\dot{k}+z_{CoM}\dot{l}}{Mg}$$

## 3. Momentum Allocation Control Framework

To walk on uneven ground, a robot has to deal with various unstable factors. For example, the landing height and inclination of the sole need to be adjusted according to the ground conditions, and the center of mass position and trunk inclination should be adjusted in real time according to the IMU. In order to achieve stable walking, the control framework needs to solve all the above problems. In this section, we will introduce the three controllers used in this paper to achieve stable uneven terrain walking.

### 3.1. Momentum Decreasing Controller

It can be seen from Equation (7) that when $x_{CoM}$ and $x_{ZMP}$ remain unchanged, the changes of $\dot{k}$ and $\dot{l}$ will affect each other. Usually, when a robot wants to change its motion state(CoM position, velocity, etc.), it need to move its ZMP. However, the ZMP position of the robot cannot be changed under certain postures or contact configurations. At this time, if the robot wants to change its motion state, it can use the momentum allocation method.

In a stationary case, we want to keep linear momentum at zero, such that the center of mass can easily stay in the support polygon. In some cases, the robot lands in an unexpected landing position, causing it to rotate around the CoP. We want to decrease the angular momentum first, then decrease the overall momentum, and finally recover body posture. This leads to two different ways of momentum allocation: linear momentum compensates for angular momentum and angular momentum compensates for linear momentum.

**Figure 2:**
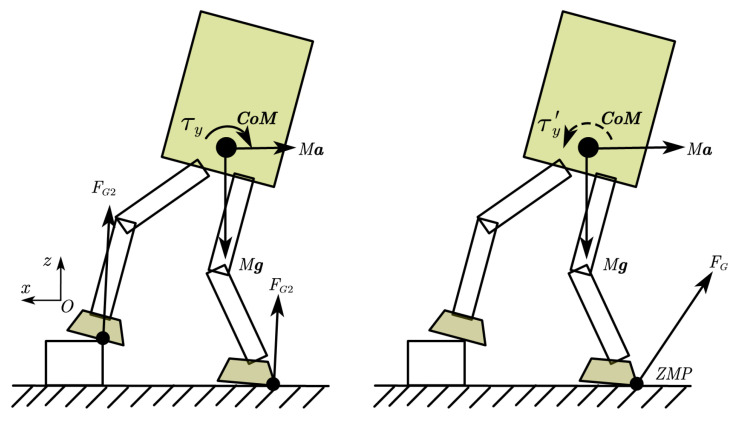
Angular momentum compensation strategy. The left side is the original situation, and the robot has a large rotation trend. On the right is the case of generating backward acceleration, which reduces the rotation trend.

As shown in Figure 2, the robot tends to rotate clockwise and fall backward. If we want to reduce the rotation trend of the robot, we can make the robot produce a backward acceleration. In this case, we can design an angular momentum-decreasing controller with a simple PD control law:

(8) x¨cmd=φl1ky−krefy+φl2x˙−x˙ref+φl3x−xref

where *x* is the measured position of the center of mass, $k_{y}$ is the measured angular momentum around the y-axis, and $\varphi_{l1}$ to $\varphi_{l3}$ is the feedback coefficient adjusted manually. The term φl1ky−krefy is to generate allocated linear momentum, and the term φl2x˙−x˙ref+φl3x−xref is to restore the robot state to the reference state; ${\ddot{x}}_{cmd}$ is integrated to obtain ${\dot{x}}_{cmd}$ and $x_{cmd}$ for inverse kinematics.

When the robot obtains a large angular momentum $k_{y}$, the controller will generate a backward acceleration to decrease it. While the angular momentum is decreasing, the controller will gradually restore CoM position because of the CoM velocity and CoM position feedback term.

**Figure 3:**
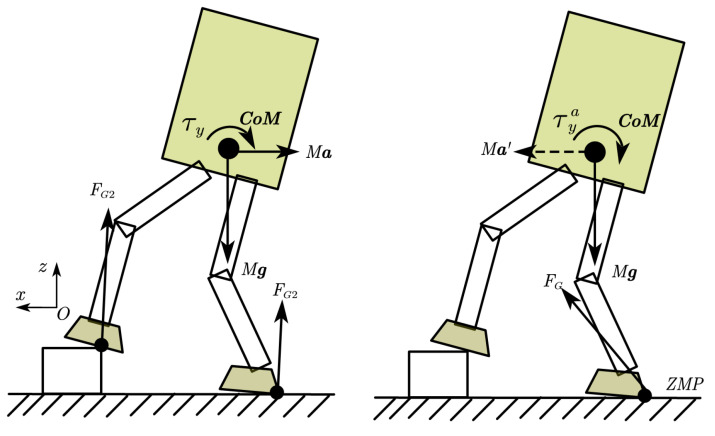
Linear momentum compensation strategy. The left side is the original situation, and the robot has a larger trend of accelerating backward. On the right is the case of generating instantaneous clockwise rotation, which reduces the trend of accelerating backward.

Similar to the case where the linear momentum compensates for the angular momentum, the robot could generate a clockwise rotational acceleration to decrease backward acceleration, as shown in Figure 3. In this case, we have another control law:

(9) θ¨cmd=φk1ly−lrefy+φk2θ˙−θ˙ref+φk3θ−θref

where θ is the measured orientation of the torso, and $l_{x}$ is the measured linear momentum along the x-axis.

Note that $\ddot{\theta }$ is the torso rotational acceleration, which differs from the angular momentum rate of change. However, unlike the relationship between the CoM acceleration and the linear momentum rate of change which could be accurately mapped, there is no specific index for the robot corresponding to the angular momentum rate of change, except for solving an optimization problem to obtain whole body posture. So in this case, we replace the angular momentum rate of change with torso rotational acceleration. Experiments show that the replacement is accurate enough for robots with large trunk and upper limb mass, for example, a proportion of trunk and upper limb mass more than 70%.

### 3.2. Posture Controller

In addition to the center of mass momentum, we also need to control the whole body posture of the robot. This is especially necessary when the height of the floor varies. We use the foot orientation to control the torso orientation. The controller is as follows: ${\Delta \theta }^{S}$ is the foot orientation compensation angle, θref is the torso orientation reference, and subscript pre represents the value of the last control period of the variable. In both pitch and roll directions, we use the same compensation method.

(10) Δθ¨S=k1θref+k2θ˙ref+k3Δθ˙preSΔθ˙S=Δθ˙preS+Δθ¨SΔtΔθS=ΔθpreS+Δθ˙SΔt

Except for foot orientation, the leg length also needs to be adjusted. We use Equation (11) to calculate the leg length compensation value, while *D* stands for the foot separation.

(11) Δh=θSD2,θS=θref+ΔθS

**Figure 4:**
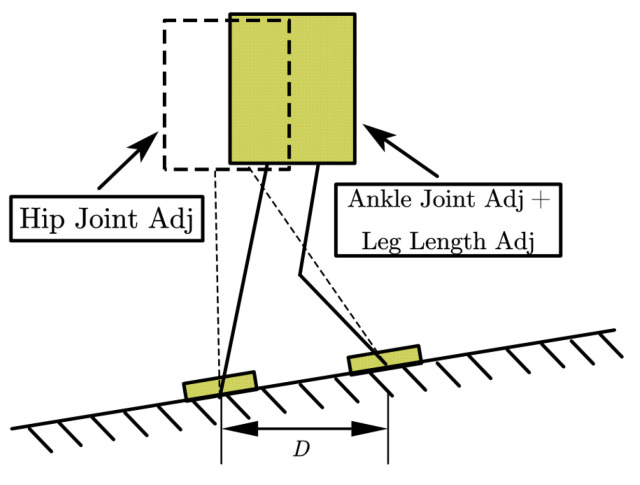
Illustration of the leg length compensation method.

The effect of leg length compensation is shown in Figure 4. We can use just roll or pitch direction foot orientation to replace $\theta^{S}$ for simplification.

### 3.3. Contact Compliance Control

Although the posture controller can restore the trunk posture to a certain extent, the robot still easily falls when a major impact suddenly occurs. When the robot’s foot touches the ground before the scheduled time or the foot steps on a protruding object, there will be a large impact produced in the contact position [25], which will significantly affect the robot’s locomotion. Therefore, the momentum allocation controller and the posture controller alone cannot keep the robot walking stably. In order to reduce the landing impact and make the landing process compliant, we used an admittance control on the sole of the robot’s feet.

**Figure 5:**
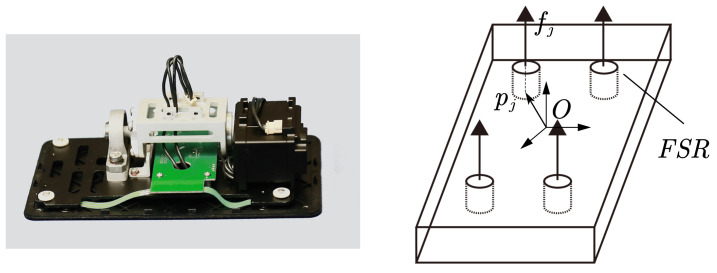
Picture of force sensitive resistor (FSR) sensors and a simplified force measuring schematic diagram.

There are four force-sensitive resistors (FSRs) in each sole, shown in Figure 5. The contact between the FSR units and the ground is regarded as point contact. The FSR unit measures the pressure between the foot and the ground at this point.

Using four FSR units, the ZMP of the sole can be calculated as follow:

(12) $$\begin{array}{c} p_{ZMP}=\frac{\sum_{j=1}^{N}p_{j}f_{j}}{\sum_{j=1}^{N}f_{j}} \end{array}$$

where $p_{j}$ is the position of the *j*th FSR unit, $f_{j}$ is the measured force, and $p_{ZMP}$ is the measured ZMP.

After obtaining the ZMP, we can apply admittance control using the following controller. Both the x-axis and y-axis can use the same control law.

(13) θ¨imp=1MpZMP−pZMPref+Bθ˙imp+Kθimpθ˙imp=θ˙preimp+θ¨impΔtθimp=θpreimp+θ˙impΔt

where *M*, *B*, and *K* are the designed control parameters, which are obtained by empirical tuning in this work, and $\theta^{imp}$ is the foot orientation compensation angle of the admittance controller.

Due to the low measurement accuracy of the FSR unit, this controller may lead to trajectory divergence when the robot is in the single support phase. So we only enable the controller for the double support phase.

### 3.4. Arm Swing Control

Due to the limited width of the robot’s sole in the Y direction, it is more difficult to maintain balance and track the CoM trajectory in the Y direction than in the X direction. In order to enhance the robot’s ability to maintain Y-direction balance, in addition to the above momentum allocation and attitude controller, we also use arm swing to adjust the position of the robot’s CoM for balance control. We use the arm swing control to adjust the CoM position rather than directly changing the trunk position for CoM adjustment because we found that adjusting the trunk position in the Y direction to achieve centroid position adjustment will bring additional unstable factors and make it easier to fall. We speculate that adjusting the centroid by changing the trunk position involves changing whole-body momentum and foot CoP, which has a negative impact on the momentum allocation and attitude control.

In the arm swing controller, we use the trunk inclination and angular velocity as inputs and use the PD controller to calculate the compensation angle of the arm swing, which is similar to the posture controller in Equation (10). Due to the small mass of both hands, the effect of the arm swing controller is not obvious, but experiments show that this method can reduce the shaking amplitude of the trunk in the Y direction and the falling probability when the robot is walking.

Note that the arm swing control mainly acts on the coronal plane, while the momentum allocation control mainly acts on the sagittal plane. The left and right swing of the arm has little impact on the momentum and angular momentum in the X direction of the robot, so the two control methods can coexist without affecting each other.

### 3.5. Control Diagram

**Figure 6:**
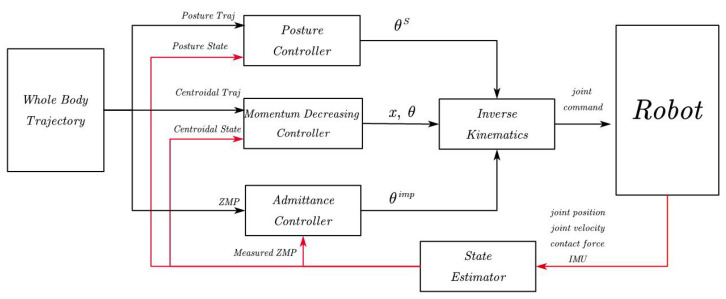
Overall control framework. In the single and double support phases, different controllers are enabled for trajectory control. The momentum decreasing controller and the admittance controller are enabled only in the double support phase. The red line represents the feedback states.

The overall control diagram is shown in Figure 6. In our experiments, we found that it is better not to control the momentum and admittance during the single support phase because in that phase the robot can only use a single sole for balance control, and it cannot achieve good momentum trajectory tracking. Therefore, in the diagram, not all controllers are enabled during the walk process. In the single support phase, only the posture controller is enabled. The double support phase is the main stage for the robot to implement momentum allocation control, so all three controllers are enabled in the double support phase.

## 4. Experiment Results and Discussion

In order to test the effectiveness of the momentum allocation control framework, we carried out three experiments: standing balancing, discrete terrain walking, and uneven terrain walking (See Supplementary Materials). Table 2 list the detailed experiment setups. For standing balancing, we conducted a simulation and a hardware test to verify the proposed methods. For discrete terrain walking and uneven terrain walking, we performed hardware tests to explore the feedback control performance of the proposed methods.

### 4.1. Standing Balancing

**Figure 7:**
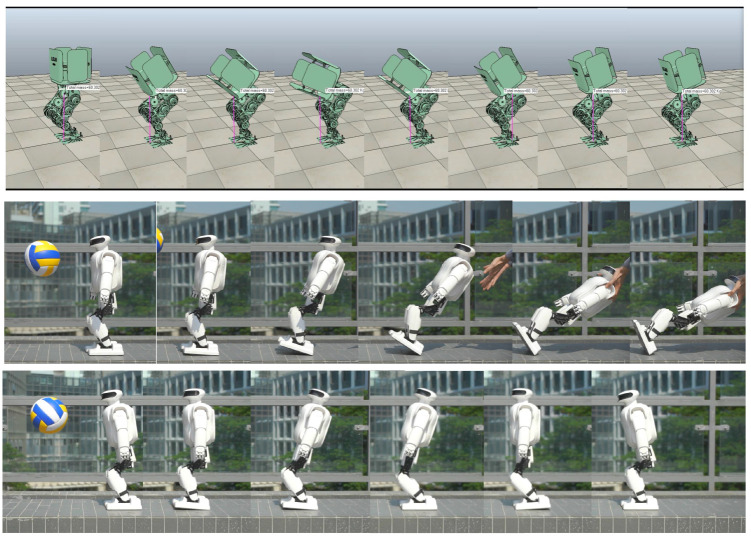
The standing balancing experiment setup. The first row is the robot falling down without momentum compensation control. The second row is the robot recovery from disturbance with momentum compensation control.

We tested the disturbance resistance of the robot in a standing state using the angular momentum compensating linear momentum method. In the simulation, a humanoid virtual prototype with a 60 kg weight and a 1.3 m height was pushed by a 400 N force lasting for 0.1 s and recovering in 2.0 s. In the hardware test, the robot was hit by a volleyball with speeds ranging from 6.5 m/s to 5.3 m/s in multiple tests. Figure 7 shows the simulation and hardware test results. Below is the detailed analysis of the hardware test.

**Figure 8:**
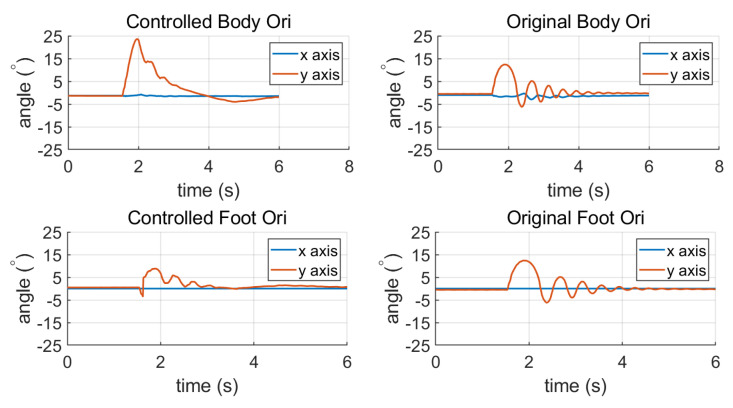
The inclination change of standing balancing. The left is the torso and sole inclination with momentum compensation, and the right is the torso and sole inclination without momentum compensation.

We can see from Figure 8 that when the momentum allocation control is adopted, the robot does not sway back and forth; instead it smoothly restores to a steady state. The max sole orientation error is decreased, indicating that the allocation control decreases the impact effect.

**Figure 9:**
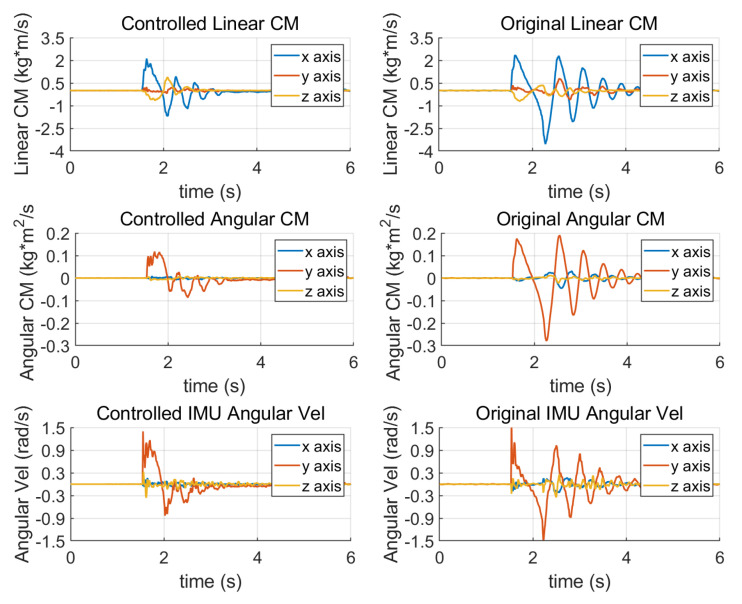
The linear and angular momentum change of standing balancing. Both the linear and angular momentum are decreased after the allocation control. The maximum angular velocity of the torso is also reduced because of the rapid stabilization process.

Figure 9 shows the linear and angular momentum changes of the robot after being hit by the volleyball. After adopting momentum allocation, the linear momentum increment of the robot is obviously reduced. As the torso rotates to generate target angular momentum, the measured angular momentum is increased, but not by much. However, the increase of integral of angular momentum is bigger. The increment of the angular momentum integral is reflected in the obvious forward tilt of the robot torso under the action of momentum allocation after being pushed. The angular velocity curve measured by IMU is consistent with the angular momentum curve, which verifies the mechanism of momentum allocation.

**Figure 10:**
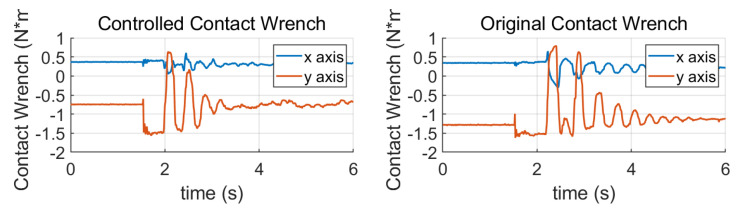
Changes of the contact wrench in standing balancing. The torque changes greatly in the Y direction of the sole.

Figure 10 shows the change of contact wrench measured by FSR unit. The contact wrench will reach the max value if the ZMP moves to the edge of the foot, which is about 1.5 Nm for Roban. We can notice that the contact wrench recovers balance faster in the controlled case. Note that when analyzing the forward walking gait, we assume the robot does not rotate fast around the Z axis, so the contact wrench in the Z axis can be ignored.

The above experiments show the effectiveness of the momentum allocation, but they also show that the momentum allocation based on angular momentum is mainly used to speed up the stable process of the robot after being pushed. However, it has a limited effect on reducing the trajectory deviation of the robot.

### 4.2. Discrete Terrain Walking

Different from the standing balancing experiment, in the discrete terrain walking experiment, we found that the swing of the trunk easily causes instability. In this case, we manually tuned and weakened the feedback coefficient of the angular momentum compensation strategy so that linear momentum compensation strategy plays a major role.

**Figure 11:**
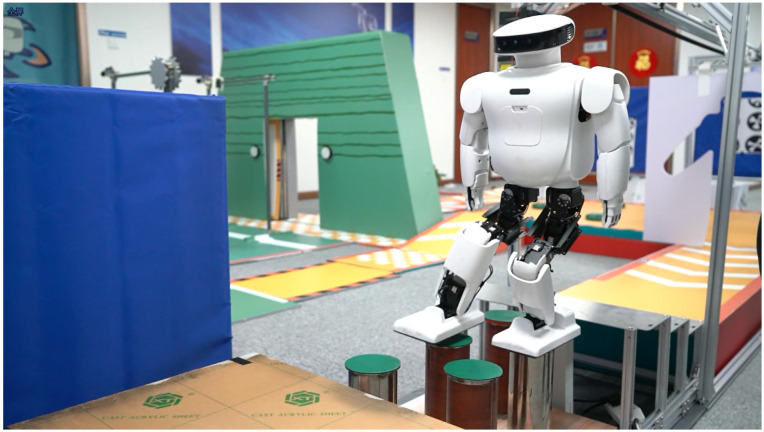
Discrete terrain walking. The walking area is limited to two rows of support columns.

We tested the discrete terrain walking control with two rows of circular support columns, as shown in Figure 11. Due to the limited contact area between the foot and the support column, the robot easily falls. Using the linear momentum compensation method, we improved the success rate of the experiment from about 40% to about 90%. The test process was to run the test program 20 times under the same start conditions.

**Figure 12:**
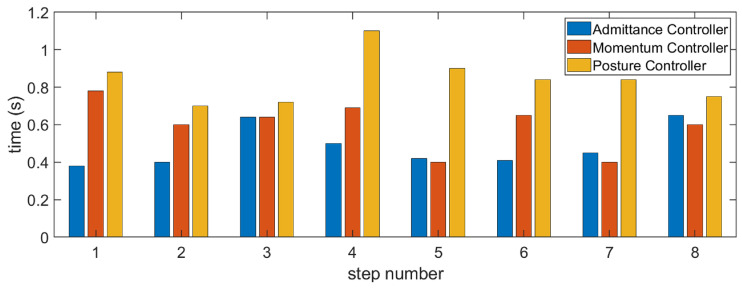
Different controller convergence times. Most of the time, the admittance controller converges first, then the momentum controller, and finally the posture controller.

We recorded the convergence time of each controller in a walking process, and obtained the histogram shown in Figure 12. It can be seen from the figure that in most cases, the admittance controller reached the stable state first; then, the momentum controller and the posture controller reached the stable state, respectively. The time distribution meets expectations.

### 4.3. Uneven Terrain Walking

When walking on uneven ground, due to the unknown terrain, the robot may have a large landing impact. When the robot steps on a high obstacle when moving forward, it causes the robot to fall backward with clockwise angular momentum, as shown in Figure 2. In order to prevent the robot from falling, the method of linear momentum compensating angular momentum was adopted for momentum allocation.

**Figure 13:**
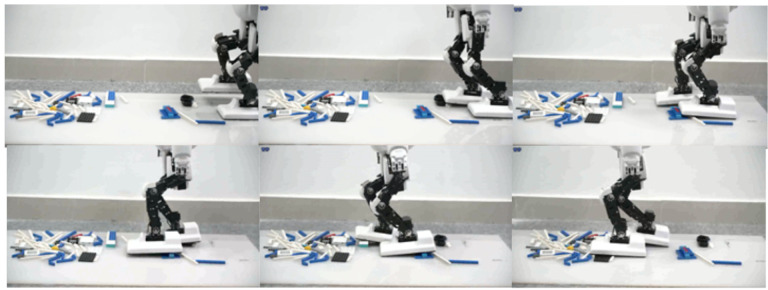
Walking on the floor covered with building blocks.

**Figure 14:**
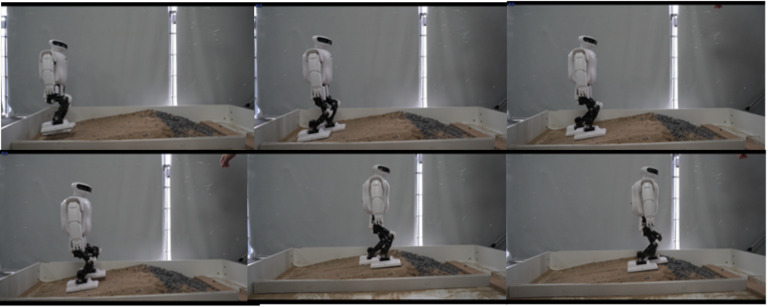
Walking on a sandy slope.

We experimented with structured uneven terrain walking and soft-ground walking. The step time cycle was about 2.0 to 3.5 s per step, which was limited by the controller convergence time and was different in every step. The step length was about 8 cm, which was limited by the kinematic constraint and walking stability and was determined empirically. The obstacle height was about 2 cm, approximately 3.3% of the total body height of 65 cm. The video frame capture of walking on structured uneven ground is shown in Figure 13 and Figure 14. In the double support phase, three controllers act simultaneously, and due to the quasi-static gait, the robot will reduce its speed to zero in this phase. When the robot steps on different ground, the interference to the robot is different, and the adjustment time is also different. Therefore, we should set up the blocking mechanism of the controller. That is, we set the control objectives for the three controllers, and only when all the controllers have completed the control objectives, is the robot’s bipedal support phase considered to be finished. Then, the next step of walking is started. The control objectives include angular momentum less than 0.05 N·m·s, trunk inclination less than 0.05 radian, and contact wrench measured with the FSR sensor less than 0.09 Nm.

### 4.4. Discussion

Based on the proposed method, three experiments were carried out: standing balancing, discrete terrain walking, and uneven terrain walking. In the standing balancing simulation, a humanoid can recover from an impact of 400 N/0.1 s. In the hardware experiment, a volleyball was used to hit the standing robot, and its anti-disturbance control ability with angular momentum compensation control was observed.

In the discrete terrain walking experiment, the robot was placed in front of multiple support columns and needed to walk through them. Although the foot of the robot is not small relative to its height, the contact area between the foot and the support column was limited. The linear momentum compensation method was implemented in the double support phase, improving the walking success rate from 40% to about 90%.

Finally, in the uneven terrain walking was carried out to test the effectiveness of the whole control framework. The robot can walk on the rugged ground with an obstacle height of about 2 cm, about 3.3% of the total body height of 65 cm. The average walking speed was 2.8 s per step, with a step length of about 8 cm. The effectiveness of the momentum allocation control was verified.

As a comparison of different humanoid prototypes, [26] shows that the German Research Foundation’s full-size humanoid robot Lola can perform fast walking of about 0.5 s per step on hard rough ground. In [27], the full-size TORO robot of Deutsches Zentrum für Luft-und Raumfahrt can walk for about 0.8∼1.5 s per step on hard and soft ground. There are few studies on child-size robots walking on rough ground. [28] shows that the robot iCub performs closed-loop walking at a speed of about 3 s per step.

From the above experiments, it can be seen that the proposed method can achieve anti-disturbance control of child-size humanoid robots and can improve the stability of walking on non-flat terrain. However, it is also found that it is difficult to improve the walking speed with the proposed method. Based on the experimental tuning process, we speculate that the possible factors limiting walking speed are: (1) the IMU sensor’s measurement bias, (2) gear backlash of joints, (3) the robot’s lower control frequency (100 Hz), and (4) the position tracking accuracy of joints. Moreover, there are three controllers in the framework, and there may be interference between them. However, in the uneven terrain walking experiment, all three controllers were necessary to prevent the robot from falling, and an experimental process that can accurately compare the effects of the three controllers is still being designed.

## 5. Conclusions

This paper studies the balance control problem when the ZMP moves to the edge of the supporting polygon, and the landing position cannot be changed. This situation often occurs in the process of complex motion of robots. Solving this problem can greatly improve the motion control effect of robots. Among the three strategies of balance control, walking strategy, hip strategy, and ankle strategy, hip strategy is the most suitable for this situation. This paper designs a simple momentum allocation method based on a PD controller, which is combined with a whole-body posture controller and a landing admittance controller to implement robot momentum allocation control. Based on the control framework, this paper verifies the effectiveness of angular momentum compensation and linear momentum compensation methods, respectively. The control framework enhances the anti-disturbance ability of the robot when standing and improves the stability in the X direction when walking on discrete and uneven ground. The above experiments verify the effectiveness of momentum allocation on the physical prototype.

This paper also has the following limitations. Firstly, there is the limitation of hardware. The limited trajectory tracking performance of the robot makes the stable convergence process slow, which leads to a slow walking speed of 2.8 s per step, with a step length of about 8 cm. Secondly, the principle of momentum allocation in this paper is to reduce the momentum and angular momentum to zero at the same time in the stable control stage of walking. This is suitable for control tasks such as intermittent walking, which has zero angular momentum. However, other control tasks such as jumping and dancing will involve non-zero angular momentum, and a more general method of momentum allocation is needed.

As for future work, we will first focus on improving the hardware experimental platform and design more detailed controller verification steps to explore the performance of the three controllers. Other research directions include the design of momentum allocation methods with non-zero angular momentum trajectory such as continuous walking, running, and dancing. It is also an interesting topic to use quadratic programming (QP) and other methods to map the allocated momentum to whole body trajectory to achieve more accurate momentum control.

## Figures and Tables

**Table 1:** Table for notations.

Notation	Description
$x_{CoM}$	The robot’s center of mass in the *x* axis
$x_{ZMP}$	The robot’s zero moment point in the *x* axis
$F_{G}$	The ground reaction force
$r_{CoM}$	The robot’s center of mass position
$\tau_{y}\left({\vec{r}}_{CoM}\right)$	Total moment acting on the CoM
*q*	Generalized position vector
*k*	Angular centroidal momentum of the robot
*l*	Linear centroidal momentum of the robot
$\theta^{T}$	The orientation of the torso
${\Delta \theta }^{S}$	The foot orientation compensate angle
${\Delta \theta }^{imp}$	Admittance controller compensate angle
$p_{ZMP}$	Measured ZMP

**Table 2:** Table for experiment setups.

Components	Characteristics
Full-size humanoid simulation model	A virtual prototype with 12 dofs
	The total height of the prototype is 1.3 m
	The total mass of the prototype is 60 kg
Child-size humanoid robot Roban	A child-size humanoid with 22 dofs
	The total height of Roban is 65 cm
	The sole length of Roban is 18 cm
	Equipped with IMU, FSR, and Joint encoders
	Able to achieve 100 Hz joint control frequency
Volleyball	A standard volleyball
	Mass of about 280 g
	Diameter of about 21 cm
Environments with support columns	Consists of five support columns
	The diameter of support column is 10 cm
	The height of support column is 30 cm
Environments with plastic building blocks	A board with plastic building blocks
	Obstacle heights range from 0.5 cm to 2.0 cm
Environments with a slope of sand	Slope made of dry sand
	The slope gradient is about 10°

## Data Availability

The data presented in this study are available on request from the corresponding author. The data are not publicly available due to operation regulations of the cooperative company.

## Supplementary Materials

The following supporting information can be downloaded at: https://www.mdpi.com/article/10.3390/s23042027/s1. Video S1: Discrete Terrain Walking; Video S2: pile; Video S3: Standing Balancing.
